# Personal

**Published:** 1895-10

**Authors:** 


### Personal.

Dr. Walter H. Kidder, for some time past assistant physician at the Buffalo state hospital, has been appointed junior assistant physician at the St. Lawrence state hospital, of which Dr. Peter M. Wise, formerly of Erie county, is superintendent. Dr. Kidder is a graduate of Buffalo High School and of the medical department of the

University of Buffalo. He is to be congratulated upon his successful competition before the civil service commission, but his removal from Buffalo is to be regretted.

---

Dr. Sherwood Dunn, formerly of this city, but for ten years past a resident of Paris, where he has been an assistant of Professor Pozzi, has taken up his residence at Los Angeles, Cal., where he will engage exclusively in the practice of gynecology and abdominal surgery.

---

Dr. William G. Bissell, of Buffalo, on October 1, 1895, will remove from No. 10 Orton place to the corner of Porter and Normal avenues, where, in his new residence, he has fitted up a private bacteriological laboratory.

---

At a recent meeting of the trustees of Jefferson Medical College, Philadelphia, the honorary degree of LL. D. was conferred on Dr. John Collins Warren, professor of surgery in Harvard university.

---

Dr. Frank J. Thornbury, of Buffalo, has removed his office to 405 Delaware avenue, near Edward street, where he will confine his practice to dermatology. Hours, 12–1 and 4–6 p. m.

---

Dr. Electa B. Whipple, of Buffalo, who has been in Europe for medical study during several months past, will return early in October and resume her professional work.

---

Dr. F. G. Moehlau, of Buffalo, has removed from 1343 Jefferson street to his new residence, No. 1300 Jefferson street. Hours, 7–9 a. m., 1–3 and 7–8 p. m.

